# Antiviral effects of ergosterol peroxide in a pig model of porcine deltacoronavirus (PDCoV) infection involves modulation of apoptosis and tight junction in the small intestine

**DOI:** 10.1186/s13567-021-00955-5

**Published:** 2021-06-14

**Authors:** Cong Duan, Junchi Wang, Yi Liu, Jialu Zhang, Jianyong Si, Zhihui Hao, Jiufeng Wang

**Affiliations:** 1 College of Veterinary Medicine, China Agricultural University, Beijing, China; 2 Institute of Medicinal Plant Development, Chinese Academy of Medical Sciences and Peking Union Medical College, Beijing, China

**Keywords:** Ergosterol peroxide, Porcine deltacoronavirus, Immunomodulatory, Antiviral, Intestinal barrier

## Abstract

Porcine deltacoronavirus (PDCoV) is a newly discovered swine enteropathogenic coronavirus with worldwide distribution. However, efficient strategies to prevent or treat the infection remain elusive. Our in vitro study revealed that ergosterol peroxide (EP) from the mushroom *Cryptoporus volvatus* has efficient anti-PDCoV properties. The aim of this study is to evaluate the potential of EP as a treatment for PDCoV in vivo and elucidate the possible mechanisms. Seven-day-old piglets were infected with PDCoV by oral administration in the presence or absence of EP. Piglets infected with PDCoV were most affected, whereas administration of EP reduced diarrhea incidence, alleviated intestinal lesion, and decreased viral load in feces and tissues. EP reduced PDCoV-induced apoptosis and enhanced tight junction protein expressions in the small intestine, maintaining the integrity of the intestinal barrier. EP showed immunomodulatory effect by suppressing PDCoV-induced pro-inflammatory cytokines and the activation of IκBα and NF-κB p65, and upregulating IFN-I expression. Knockdown of p38 inhibited PDCoV replication and alleviated PDCoV-induced apoptosis, implying that EP inhibited PDCoV replication and alleviated PDCoV-induced apoptosis via p38/MAPK signaling pathway. Collectively, ergosterol peroxide can protect piglets from PDCoV, revealing the potential of EP for development as a promising strategy for treating and controlling the infection of PDCoV.

## Introduction

Porcine deltacoronavirus (PDCoV) is a swine enteropathogenic coronavirus (CoV) that can cause severe dehydration, vomiting and watery diarrhea in piglets, and belongs to the genus *Deltacoronavirus* of the family *Coronaviridae* [1]. Since its first outbreak in the United States in 2014 [2], PDCoV has rapidly spread to many countries, including South Korea, Japan, Thailand and China [3–6]. PDCoV can infect a variety of animals, including calves and poultry [7, 8], highlighting its ability to cross interspecies barriers. However, effective therapeutics or vaccines to control PDCoV infection are still limited, emphasizing the urgent need for the development of drugs against PDCoV.

Our recent study has shown that ergosterol peroxide (EP) from the mushroom *Cryptoporus volvatus* possesses efficient anti-PDCoV properties [9]. It can reduce LLC-PK1 cells apoptosis caused by PDCoV infection. Apoptosis is a double-edged sword mechanism, which is used by the host to eliminate viruses and is manipulated by the virus to cause cytopathic effects in vitro and/or tissue damage in vivo [10]. The massive apoptosis of enterocytes affects the integrity of the intestinal barrier [11]. The intestinal barrier is vital in maintaining selective gut permeability to allow for nutrient absorption and preventing pathogens from entering the blood circulation. The alteration of the intestinal barrier function is believed to be involved in the pathogenesis of gut diseases. Tight junction (TJ) proteins (including claudin-1, occludin and ZO-1) comprise another mechanism that maintains the integrity of the intestinal barrier [11]. As far as we know, there is no relevant reports on the effect of PDCoV on TJ proteins in piglets. Thus, the effects of EP on modulating PDCoV-induced apoptosis and the impact of PDCoV on TJ proteins need to be elucidated in vivo.

T lymphocytes are central players in orchestrating immune responses. CD4^+^ T cells can promote effective immunity protection through direct effector functions and by helping other leukocytes to maximize the protective activities [12]. The activated CD4^+^ T cells are tailored to different types of pathogens through differentiation into functionally distinct subsets of effector T cells (Th1, Th2, and Th17) defined by expression of key transcription factors [13]. When responding to viral infection, the CD4^+^ T cells mainly have a Th1-type phenotype expressing T-bet and produce large amounts of IFNγ [12]. However, little is known about the changes of CD4^+^ T-bet^+/−^ IFNγ^+/−^ T cells after PDCoV infection.

The NF-κB signaling pathway is an evolutionarily conserved pathway that regulates a variety of physiological and pathological processes, functioning as a crucial coordinator of inflammatory and immune response [14]. NF-κB is highly activated in diverse CoVs and the NF-κB-induced inflammatory response plays an important role in the development of pathogenesis and disease in CoVs infections [15, 16]. PDCoV infection induces NF-κB activation and cytokine expression, which can be alleviated with the addition of EP in vitro [9]. Here, we detected whether there were similar phenomena in vivo.

Because viruses entirely depend on host cells to complete their life cycle, they coevolve with the host to adjust preexisting intracellular signal transduction networks to benefit their own multiplication [17, 18]. The p38 mitogen-activated protein kinase (MAPK) pathway is a well-known signal transducer that responds to extracellular stimulation by cytokines, stress, and viral infection, and in turn regulates cell differentiation, survival, and apoptosis [19]. Our previous study showed that p38 inhibitor inhibited PDCoV replication and EP alleviated p38 activation induced by PDCoV infection in vitro [9].

Herein, we characterized the inhibitory effects of EP against PDCoV in vivo. We hypothesized that apoptosis, TJ proteins, NF-κB and p38/MAPK signaling pathways in the small intestine would be involved in the mechanisms by which EP alleviated PDCoV-associated pathological manifestation in piglets. A PDCoV infection model using LLC-PK1 cells was also established to address the role of p38/MAPK signaling pathway in the anti-PDCoV effects of EP.

## Materials and methods

### Preparation of EP

Ergosterol peroxide was extracted from *Cryptoporus volvatus* with a purity of over 97%. Extraction and purity determination were done as previously described [9]. Briefly, air-dried fruiting bodies of *C. volvatus* (4800 g) were smashed and extracted with 90% ethanol 3 times under reflux for 1 h. The solvent was removed under reduced pressure to yield ~1240 g of the ethanol extract, which was then suspended in water and partitioned with petroleum ether, dichloromethane, ethyl acetate and *n*-butanol. The dichloromethane fraction (1000 g) was separated into eleven fractions (I–XI) by silica gel chromatography and eluted using dichloromethane-methanol (1:0–0:1, v/v). Fraction I (8.8 g) was then subjected to silica gel chromatography and eluted with petroleum ether-ethyl acetate (1:0–0:1, v/v) to obtain about 120 mg EP.

### Virus strain

The PDCoV CHN-HN-1601 strain (GenBank accession no: MG832584) was used in the study.

### Animals and experimental groups

Ethical committee number for the study: CAU20190816-1. All animal experimental procedures were performed in accordance with protocols approved by the Institutional Animal Care and Use Committee (IACUC) of China Agricultural University. In vivo study of piglets was performed as previously described [20]. Studies have shown that piglets around one week of age are susceptible to PDCoV [6, 21]. Therefore, a total of 15 7-day-old (Landrace × Large White) male piglets (3.0 ± 0.2 kg) were obtained from a regular commercial farm in Tianjin, China. Prior to the start of the trial, no clinical signs of diarrhea or other diseases were observed in any of the piglets, and the rectal swabs were confirmed negative for the major porcine enteric viruses (PDCoV, porcine epidemic diarrhea virus (PEDV), transmissible gastroenteritis virus (TGEV), rotavirus) by Reverse transcription PCR. On day 0, the piglets were randomly divided into the following three groups (*n* = 5 per group): (i) control group (CN), oral administration of 5 mL MEM at 8:00 am on days 1–3; (ii) PDCoV group, oral administration of 5 mL MEM containing a total of 1 × 10^6^ TCID_50_ of the PDCoV CHN-HN-1601 strain at 8:00 am on day 1, 5 mL MEM at 8:00 am on days 2–3; (iii) PDCoV + EP group, oral administration of 5 mL MEM containing a total of 1 × 10^6^ TCID_50_ of the PDCoV CHN-HN-1601 strain and EP (2.5 mg/kg body weight) at 8:00 am on day 1, 5 mL MEM containing EP (2.5 mg/kg body weight) on days 2–3. On day 5, piglets from each group were necropsied, and different tissue samples and blood samples were collected.

After infection, the piglets were observed daily for clinical signs, including vomiting, diarrhea and lethargy. Rectal temperature was measured twice daily, at 7:30 am and 7:30 pm on days 0–4. Fecal samples were collected from day 0 to day 4 to evaluate viral shedding. Severity of diarrhea was scored according to previously described with six criteria [22]: 1, hard and formed pellets; 2, non-formed pellets; 3, soft feces; 4, very soft and containing a small amount of water-like feces; 5, semisolid containing more than half water-like feces; and 6, water-like feces. Piglets were considered to have severe diarrhea when the score was 5 or 6.

### Differential blood leukocyte count

The total peripheral blood leukocyte count and population distribution was determined using a semiautomated blood cell counter (Sysmex XN-1000 V, Japan). The proportions of neutrophils, lymphocytes and monocytes were expressed as a percentage of the total number of leukocytes.

### Histological and immunofluorescent staining

Tissue samples were fixed in 10% formalin, and then dehydrated in graded ethanol, embedded in paraffin, cut in 5-μm sections, and mounted onto glass slides. After the sections were deparaffinized, rehydrated, and stained with hematoxylin and eosin (H&E), the slides were examined and analyzed with conventional light microscopy (Olympus IX71, Japan). Inflammation score was as previously described [22], with the modification including six criteria: epithelial integrity (0 = no change, 1 = shedding of < 10 epithelial cells per lesion, 2 = shedding of 11–20 epithelial cells per lesion, 3 = epithelial ulceration); central lacteal expansion (0 = no change, 1 = mild, 2 = moderate, 3 = profound); leukocyte infiltration (0 ≤ 10 leukocytes per field, 1 = 11–15 leukocytes per field, 2 = 16–20 leukocytes per field, 3 ≥ 20 leukocytes per field); submucosal edema (0 = no change, 1 = mild, 2 = moderate, 3 = profound); mucosal hyperemia (0 ≤ 10 erythrocyte per field, 1 = 11–15 erythrocyte per field, 2 = 16–20 erythrocyte per field, 3 ≥ 20 erythrocyte per field); and Peyer’s patch lesions (0 = no change, 1 = mild, 2 = moderate, 3 = profound). The summation of the scores for each parameter provides an overall inflammation score for each sample, with a range of 0–18. The typical features associated with each grade of inflammation were as follows: 0, normal; 1–5, mild inflammation; 6–12, moderate inflammation; and 13–18, severe inflammation. No less than three separate sections of each sample were examined. The scoring was performed in a blinded manner. The total ileal score for each group was calculated as the average of the scores across all animals.

For immunofluorescent staining, the sections were permeabilized with 0.2% Triton X-100 in PBS for 10 min, and blocked with 1% bovine serum albumin (BSA) for 45 min at 37 °C. Subsequently, the sections were stained with anti-PDCoV monoclonal antibody (1:1000, Medgene, USA) overnight at 4 °C. RBITC-conjugated goat anti-mouse IgG (1:200, Solarbio, China) was used as secondary antibody, and DAPI (Sigma-Aldrich, Germany) was used for nuclear staining. The slides were visualized and photographed using a Leica SP8 Laser Scanning confocal microscope (Leica Microsystems, Germany).

### RNA extraction and quantitative reverse transcription PCR (RT-qPCR)

Total RNA from piglet feces and tissue samples was extracted using the BIOG RNA Stool Kit (Bio-generating, China) and the TRIzol reagent (Invitrogen, USA) according to the manufacturer’s instructions, respectively. RNA was converted to cDNA using the HiFiScript cDNA Synthesis Kit (CoWin Biosciences, China). Viral RNA and cytokine mRNA were analyzed by RT-qPCR as previously described [9]. The specific primers crossing PDCoV ORF1a and ORF1b and the specific primers for porcine IL-1β, IL-6, IL-12, TNF-α, IFN-α, IFN-β and β-actin were listed in Table 1.

**Table 1:** **Sequences of the primers used for RT-qPCR**.

Primers name	Direction^a^	Sequence (5′ → 3′)
PDCoV	F	ACCTGTTTCCTTCGCCTTGA
	R	ACAGCGTCTGGTTGAGTTCC
IL-1β	F	AACGTGCAGTCTATGGAGT
	R	GAACACCACTTCTCTCTTCA
IL-6	F	CTGGCAGAAAACA ACCTGAACC
	R	TGATTCTCATCAAGCAGGTCTCC
IL-12	F	CGTGCCTCGGGCAATTATA
	R	CGCAGGTGAGGTCGCTAGTT
TNF-α	F	AACCTCAGATAAGCCCGTCG
	R	ACCACCAGCTGGTTGTCTTT
IFN-α	F	TCTCATGCACCAGAGCCA
	R	CCTGGACCACAGAAGGGA
IFN-β	F	AGTGCATCCTCCAAATCGCT
	R	GCTCATGGAAAGAGCTGTGGT
β-actin	F	TGACTGACTACCTCATGAAGATCC
	R	TCTCCTTAATGTCACGCACGATT

^a^F = forward; R = reverse.

### Enzyme-linked immunosorbent assay (ELISA)

Porcine-specific commercially available ELISA kits were used to measure the serum concentrations of TNF-α and IFN-γ (R&D Systems, USA).

### Flow cytometry

Peripheral blood lymphocytes were isolated using Lymphocyte Separation Solution (TBD Science Inc., China) according to the manufacturer’s instructions. The monoclonal antibodies used were as follows: mouse anti-pig CD3ε (clone P2G10, FITC-conjugated, 559582; BD Biosciences), mouse anti-pig CD4α (clone 74-12-4, PerCP-Cy5.5-conjugated, 561474; BD Biosciences), mouse anti-human T-bet (clone O4-46, phycoerythrin [PE]-conjugated, 561268; BD Biosciences), and mouse anti-pig IFN-γ (clone P2G10, AlexaFluor 647-conjugated, 561480; BD Biosciences). The stained cells were analyzed on a FACScalibur™ flow cytometer (BD Biosciences), and data analysis was performed using FlowJo software (Tree Star).

### Terminal deoxynucleotidyl transferase-mediated dUTP nick end labelling (TUNEL) assay

Paraffin-embedded intestinal tissues were evaluated using a TUNEL assay kit (Beyotime, China) for apoptosis according to the manufacturer’s instructions and examined using a Leica SP8 Laser Scanning confocal microscope (Leica Microsystems, Germany).

### Small interfering RNA (siRNA) transfection

Specific porcine p38 siRNA sequences were designed using the sequence of Sus scrofa p38 mRNA (GenBank Accession No. XM_003356616.1) and synthesized by Genepharma (Shanghai, China). NC siRNA (5′-UUCUCCGAACGUGUCACGUTT-3′) was purchased from Genepharma. The p38 siRNA sequences were as follows: 5′-CCGAGGUCUCAAGUAUAUATT-3′ (#1), 5′-GGGCAGAUCUGAACAACAUTT-3′ (#2) and 5′-GCAGGAGCUGAACAAGACATT-3′ (#3). The siRNAs were transfected using HiPerFect Transfection Reagent (Giagen, Germany) at a final concentration of 60 nM according to the manufacturer’s instructions. After 24 h, the efficiency of the siRNA was characterized by Western blot.

### Cell culture

LLC-PK1 cells (ATCC CL-101) were obtained from the American Type Culture Collection (ATCC) and cultured in MEM (Gibco, USA) supplemented with 1% antibiotic–antimycotic (Gibco, USA), 1% HEPES (Gibco, USA), 1% MEM non-essential amino acids solution (NEAA) (Gibco, USA) and 10% heat-inactivated fetal bovine serum (FBS) (Gibco, Australia) at 37 °C with 5% CO_2_. LLC-PK1 cells grown in 6-well plates were transfected with p38 siRNA #1 or NC siRNA. At 24 h post-transfection, the cells were mock-infected or infected with PDCoV (MOI = 2) in the absence or presence of EP (150 μM). After PDCoV adsorption for 1 h, the cells were further cultured in fresh medium in the absence or presence of EP (150 μM). The cells were harvested for subsequent analysis at 24 hours post-infection (hpi).

### Western blot

Intestinal tissue sample weighing 0.1 g was lysed in 1 mL radioimmunoprecipitation assay (RIPA) lysis buffer (CoWin Biosciences, China) with 1 mM phenylmethyl sulfonyl fluoride (PMSF, Beyotime, China) and 20 μM NaF for 10 min. The tissue lysates were centrifuged at 12 000 × *g* for 15 min at 4 °C to remove insoluble material, and protein concentration of the resulting supernatants was quantified using a BCA protein assay kit (Thermo Fisher Scientific, USA). The LLC-PK1 cell monolayers were lysed in the same lysate for 10 min and protein concentration was quantified using the same method. An equal amount of protein samples (20 μg) was used for Western blot assay. Primary antibodies were anti-PDCoV N (1:5, provided by Professor Pinghuang Liu, China Agricultural University), anti-phospho-NF-κB p65, anti-phospho-p38 (1:500, Cell Signaling Technology, USA), anti-phospho-IκBα (1:200, Cell Signaling Technology, USA), anti-IκBα, anti-NF-κB, anti-p38 (1:1000, Cell Signaling Technology, USA), anti-claudin-1, anti-occludin, anti-ZO-1, anti-Bax, anti-Bcl-2 (1:1000, Abcam, UK), anti-cleaved caspase-3, anti-caspase-3 (1:500, Abcam, UK) and anti-β-actin (1:1000, ProteinTech Group, USA). Horseradish peroxidase conjugated to AffiniPure goat anti-mouse IgG (1:5000, ProteinTech Group, USA) or goat anti-rabbit IgG (1:5000, ProteinTech Group, USA) were used as secondary antibodies.

### Statistical analysis

Statistical analysis was performed using IBM SPSS Statistics 25 software (IBM, USA). Differences between means were compared using Tukey’s honestly significant difference post-hoc test. The data were visualized using GraphPad Prism 7 software (GraphPad Software, USA) and expressed as mean ± SEM. The statistically significant differences were set at *P* < 0.05. *P < 0.05; **P < 0.01; ***P < 0.001.

## Results

### Ergosterol peroxide alleviated PDCoV-associated pathological manifestation in piglets

**Figure 1:**
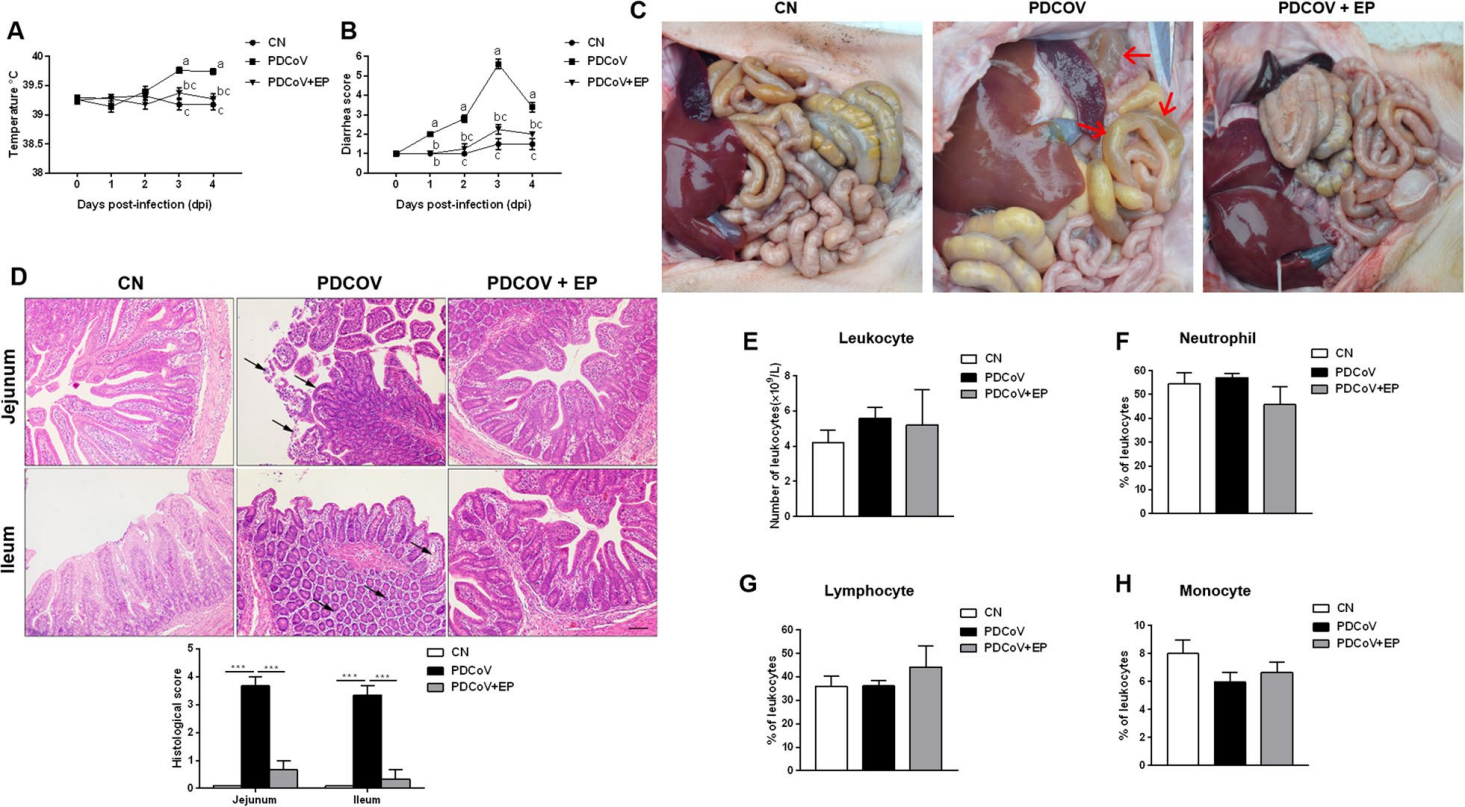
**Effect of EP on PDCoV-associated clinical signs in piglets.** Rectal temperature (**A**) and diarrhea score (**B**) of piglets that received MEM on days 1–3 (CN), MEM containing a total of 1 × 10^6^ TCID_50_ of the PDCoV CHN-HN-1601 strain on day 1 and MEM at 8:00 am on days 2–3 (PDCoV), or MEM containing a total of 1 × 10^6^ TCID_50_ of the PDCoV CHN-HN-1601 strain and EP (2.5 mg/kg body weight) on day 1 and MEM containing EP (2.5 mg/kg body weight) on days 2–3 (PDCoV + EP). Rectal temperature and severity of diarrhea were recorded daily from 0 dpi (the day before PDCoV infection) to 4 dpi. **C** Macroscopic lesions at 5 dpi. Small intestines of group PDCoV were clearly transparent, thin-walled, and a large amount of yellow fluid accumulated in the intestinal lumen (arrows). **D** Representative photomicrographs of hematoxylin and eosin-stained jejunal and ileal sections (up) and histological scores of jejunal and ileal tissues (down). Jejunum of group PDCoV showed intestinal villus blunting and rupture, enterocyte attenuation, intestinal epithelial cell shedding (arrows). Ileum of group PDCoV showed mucosal layer edema, atrophy of intestinal glands and basement membrane rupture (arrows). Peripheral blood samples of the indicated groups were collected at 5 dpi. Shown are results for total peripheral blood leukocyte count (**E**) and the percentage of neutrophils (**F**), lymphocytes (**G**) and monocytes (**H**) of the total number of peripheral blood leukocytes. Data are presented as the mean ± SEM. Mean values at the same time point without a common superscript (a, b, c) differ significantly (*P* < 0.05). ***P < 0.001. Scale bars, 200 μm.

Before infection, rectal temperature was within the normal range (39.27 °C ± 0.13 °C) for all the three groups (Figure 1A). However, on 3 dpi, the rectal temperature of group PDCoV increased to 39.76 °C ± 0.16 °C, which was significantly higher than that of groups CN (*P* < 0.001) and PDCoV + EP (*P* = 0.009), and this trend continued until 4 dpi. No significant differences were observed between groups CN and PDCoV + EP.

Following PDCoV infection, all piglets in group PDCoV exhibited diarrhea lasting to 4 dpi, especially on 3 dpi, presenting watery diarrhea (Figure 1B). Administration of EP reduced diarrhea incidence to a level similar to that of group CN. Besides, only the piglets in group PDCoV exhibited lethargy and anorexia on 2 days post-infection (dpi) to 4 dpi, and two of them vomited on 2 dpi to 3 dpi. Post-mortem examination showed that the small intestines of group PDCoV were clearly transparent, thin-walled, and a large amount of yellow fluid accumulated in the intestinal lumen (Figure 1C). No lesions were observed in groups CN and PDCoV + EP at necropsy (Figure 1C). Histopathologic analysis revealed that the jejunum and ileum of group PDCoV exhibited obvious pathological changes (Figure 1D). Lesions of jejunum showed intestinal villus blunting and rupture, enterocyte attenuation, intestinal epithelial cell shedding. Lesions of ileum showed mucosal layer edema, atrophy of intestinal glands and basement membrane rupture. In comparison, only slight changes were detected in group PDCoV + EP (Figure 1D), presenting mild hyperemia in the jejunum and ileum. No lesions were seen in group CN (Figure 1D).

PDCoV infection had no significant effects on the total peripheral blood leukocyte count (Figure 1E), and the percentage of neutrophils (Figure 1F), lymphocytes (Figure 1G) and monocytes (Figure 1H) compared with group CN.

### Administration of EP inhibited PDCoV replication in piglets

**Figure 2:**
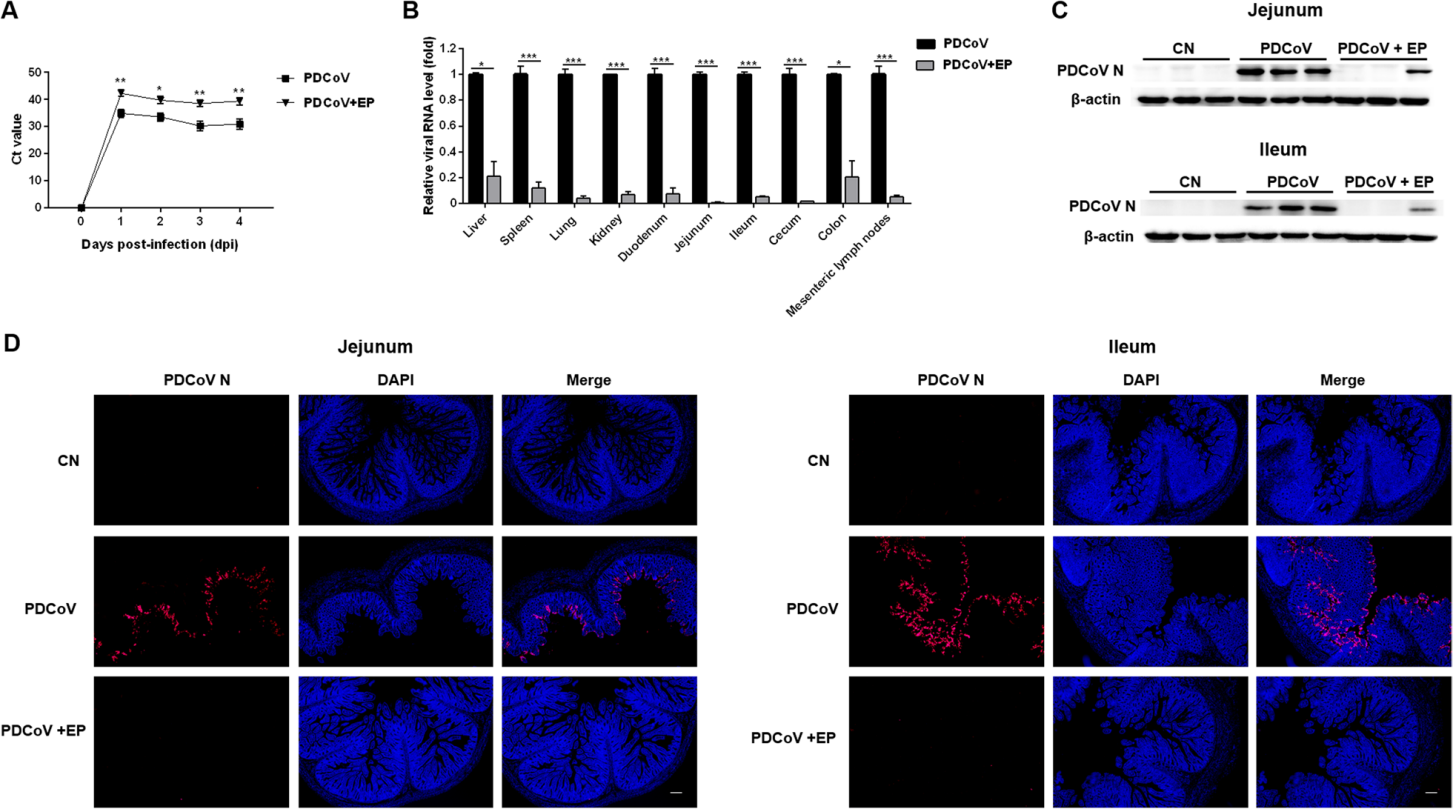
**Antiviral effect of EP on PDCoV replication in piglets**. **A** Effect of EP on fecal viral shedding. Fecal samples of the indicated groups were collected from 0 dpi (the day before PDCoV infection) to 4 dpi. The relative viral RNA level in the fecal samples was analyzed by RT-qPCR. **B** Effect of EP on virus distribution. Different tissue samples of the indicated groups were collected at 5 dpi. The relative viral RNA level in the collected samples was analyzed by RT-qPCR. **C** Western blot analysis of proteins from jejunal and ileal tissues probed with the anti-PDCoV N antibody. The loading was normalized with β-actin. **D** Fluorescence imaging of jejunal and ileal tissues. Red fluorescence represents the PDCoV distribution, and blue fluorescence represents the nuclear distribution. Scale bars, 20 μm. Data are presented as the mean ± SEM. *P < 0.05; **P < 0.01; ***P < 0.001.

To examine the inhibitory effect of EP on PDCoV replication in vivo, we determined the fecal viral shedding and virus distribution in piglets. Compared with group PDCoV, group PDCoV + EP had lower levels of viral RNA in the fecal samples (Figure 2A). Administration of EP also reduced PDCoV viral load in various tissues (Figure 2B). The relative viral RNA levels in the liver (*P* = 0.010), spleen (*P* < 0.001), lung (*P* < 0.001), kidney (*P* < 0.001), duodenum (*P* < 0.001), jejunum (*P* < 0.001), ileum (*P* < 0.001), cecum (*P* < 0.001), colon (*P* = 0.015), and mesenteric lymph nodes (MLN) (*P* < 0.001) in group PDCoV + EP were significantly lower than those in group PDCoV (Figure 2B). No PDCoV RNA was detected in group CN. These results were further confirmed in the jejunum and ileum by Western blot analysis (Figure 2C) and fluorescence imaging (Figure 2D). Western blot analysis showed that the PDCoV N protein expression in the jejunum and ileum of group PDCoV + EP was remarkably lower than that of group PDCoV (Figure 2C). Fluorescence imaging of the jejunum and ileum demonstrated that numerous PDCoV antigen-positive cells were observed in group PDCoV, while PDCoV antigen-positive cells were hardly observed in group PDCoV + EP (Figure 2D).

### Administration of EP suppressed PDCoV-induced apoptosis in the small intestine

**Figure 3:**
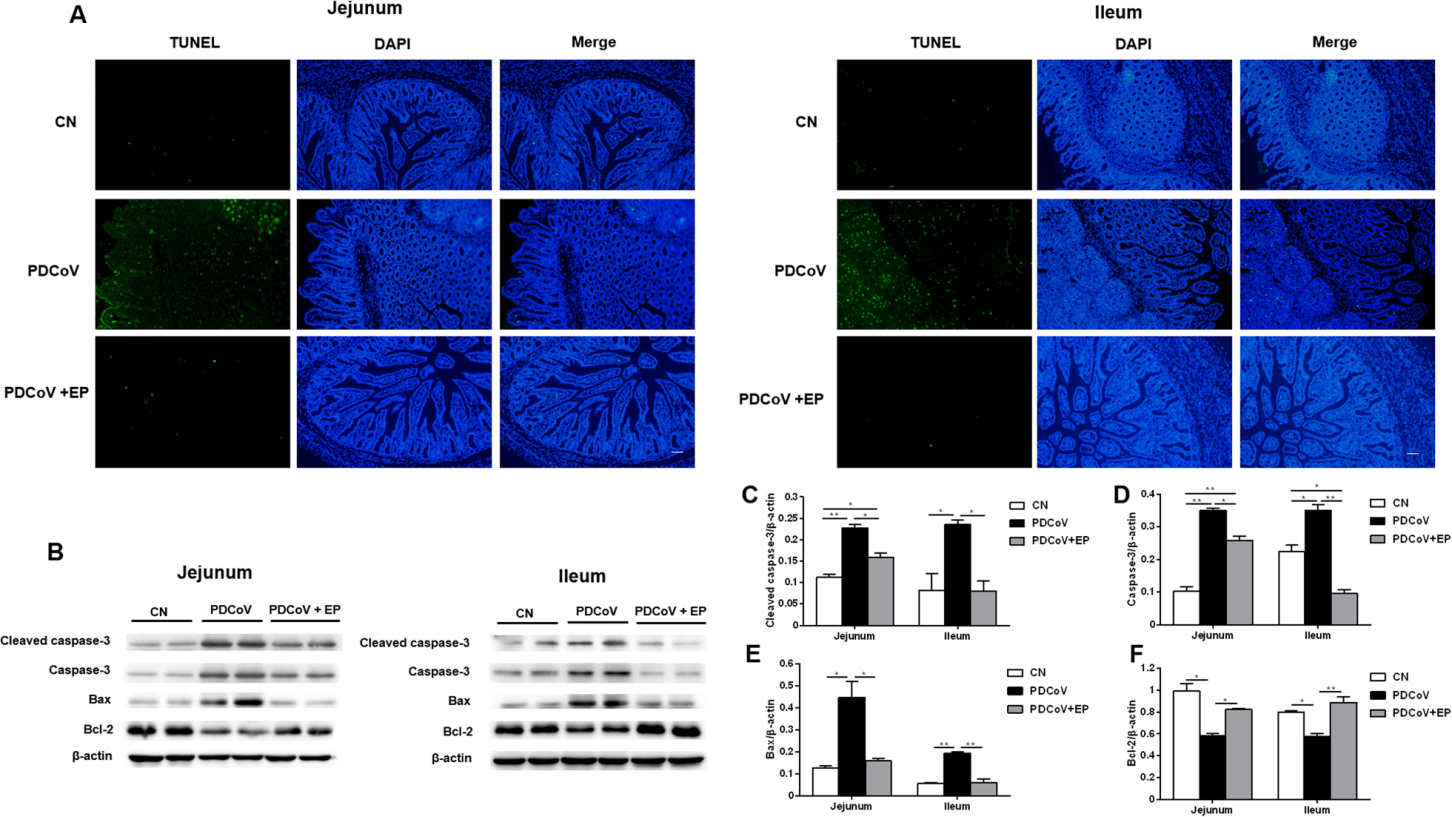
**Effect of EP on PDCoV-induced apoptosis in the small intestine.** Intestinal tissue samples of the indicated groups were collected at 5 dpi. **A** TUNEL assay of paraffin-embedded jejunal and ileal tissues. Green fluorescence represents the TUNEL-positive (apoptotic) cells, and blue fluorescence represents the nuclear distribution. Scale bars, 20 μm. **B** Western blot analysis of proteins from jejunal and ileal tissues probed with the anti-cleaved caspase-3, anti-caspase-3, anti-Bax and anti-Bcl-2 antibody. **C**–**F**. Results are presented as the ratio of target protein band intensity to β-actin band intensity. Data are presented as the mean ± SEM. *P < 0.05; **P < 0.01.

A great number of TUNEL-positive cells were detected in the jejunum and ileum of group PDCoV (Figure 3A), indicating that plentiful immune cells or enterocytes underwent apoptosis. However, only few TUNEL-positive cells were occasionally detected in groups CN and PDCoV + EP. These results were further confirmed by the analysis of the main apoptotic executioner caspase-3, pro-apoptotic Bax and anti-apoptotic Bcl-2 protein expression in the small intestine of piglets. PDCoV infection increased the expressions of cleaved caspase-3 (Figures 3B, C; *P* = 0.003 and *P* = 0.046, respectively) and Bax (Figures 3B, E; *P* = 0.026 and *P* = 0.003, respectively), and decreased expression of Bcl-2 (Figures 3B, F; *P* = 0.011 and *P* = 0.020, respectively) in the jejunum and ileum compared with group CN. These changes were attenuated by oral administration of EP. Lower expressions of cleaved caspase-3 (Figures 3B, C; *P* = 0.015 and *P* = 0.044, respectively) and Bax (Figures 3B, E; *P* = 0.033 and *P* = 0.004, respectively), and higher expression of Bcl-2 (Figures 3B, F; *P* = 0.047 and *P* = 0.008, respectively) were observed in the jejunum and ileum of group PDCoV + EP compared with group PDCoV.

### Administration of EP enhanced claudin-1 and ZO-1 expression in the small intestine

**Figure 4:**
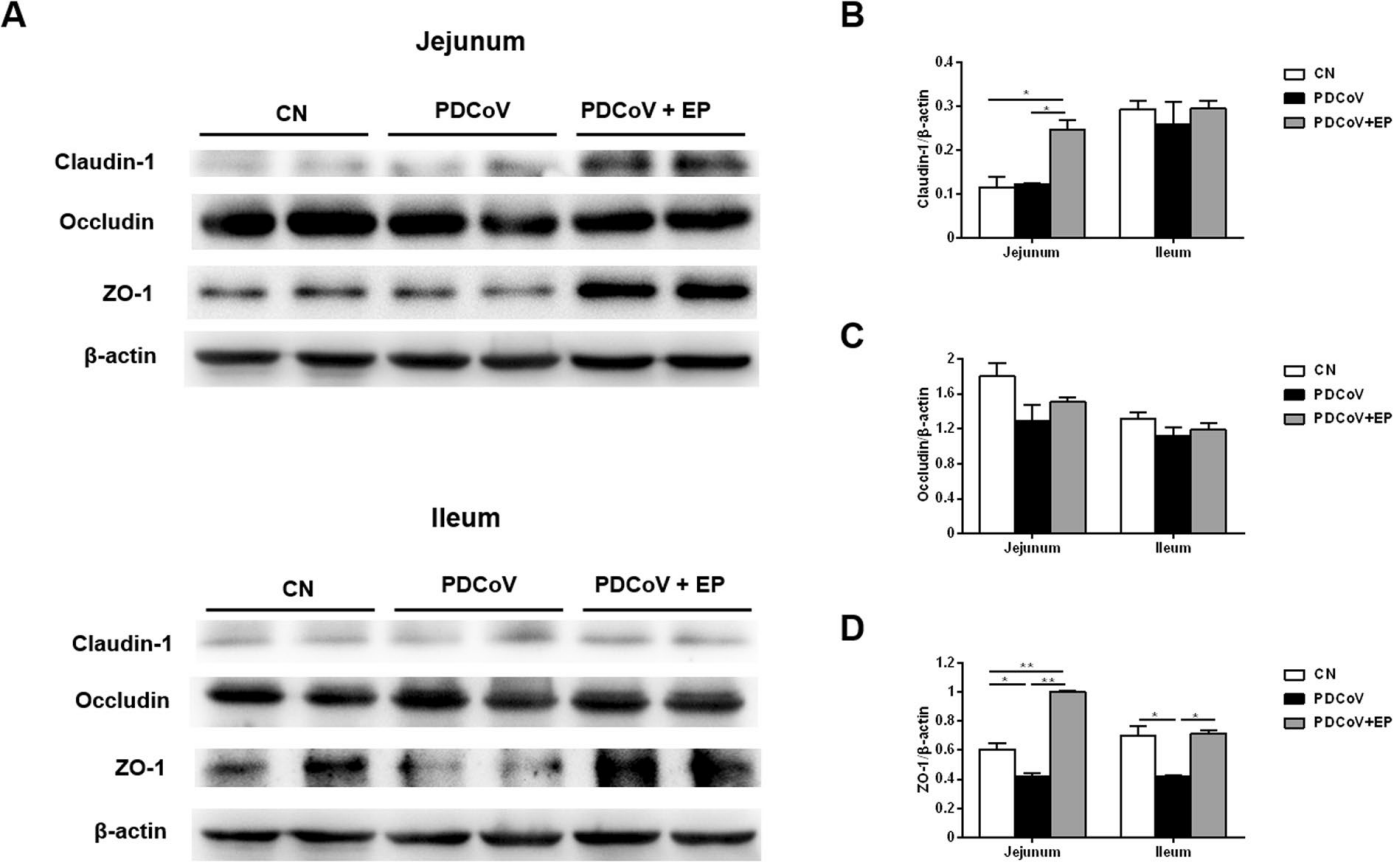
**Effect of EP on tight junction proteins in the small intestine.** **A** Western blot analysis of proteins from jejunal and ileal tissues probed with the anti-claudin-1, anti-occludin and anti-ZO-1 antibody. **B**–**D**. Results are presented as the ratio of target protein band intensity to the intensity of the β-actin band. Data are presented as the mean ± SEM. *P < 0.05; **P < 0.01.

To detect the impact of PDCoV infection on TJ proteins and the regulation of EP on them, the expressions of claudin-1, occludin, and ZO-1 in the small intestine were analyzed by Western blot. The claudin-1 expression in the jejunum was higher in group PDCoV + EP than in groups CN and PDCoV (Figures 4A, B; *P* = 0.022 and *P* = 0.025, respectively). No differences were found in the jejunum and ileum between groups CN and PDCoV. The occludin expression in the jejunum and ileum had no difference among three groups (Figures 4A, C). The ZO-1 expression in the jejunum and ileum was lower in group PDCoV than in group CN (Figures 4A, D; *P* = 0.044 and *P* = 0.034, respectively). Compared with group PDCoV, administration of EP increased the expression of ZO-1 in the jejunum and ileum of group PDCoV + EP (Figures 4A, D; *P* = 0.002 and *P* = 0.030, respectively).

### PDCoV infection increased the percentage of CD4^+^ T cells but decreased the percentages of CD4^+^ T-bet^+^ T cells and CD4^+^ T-bet^+^ IFNγ^+^ T cells in peripheral blood

**Figure 5:**
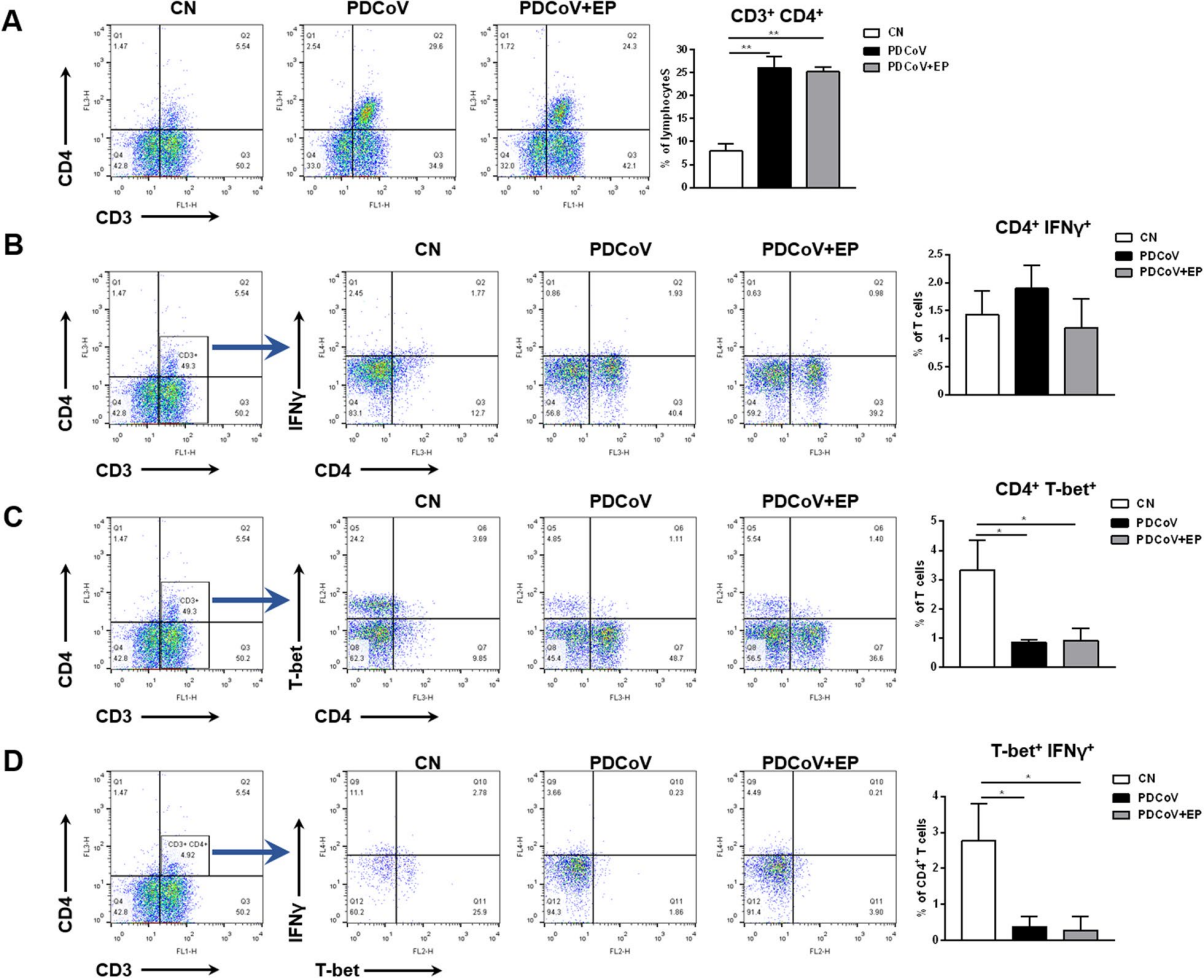
**Effect of PDCoV infection on T-lymphocyte subpopulations in the peripheral blood.** Peripheral blood samples of the indicated groups were collected at 5 dpi. Left, representative flow cytometry dot plots show the percentage of CD4^+^ T cells within the peripheral blood lymphocytes (**A**), the percentage of CD4^+^ IFNγ^+^ T cells (**B**) and CD4^+^ T-bet^+^ T cells (**C**) within the peripheral blood T cells, and the percentage of CD4^+^ T-bet^+^ IFNγ^+^ T cells within the peripheral blood CD4^+^ T cells (**D**) in the indicated piglets. Right, flow cytometry analysis of the percentage of CD4^+^ T cells within the peripheral blood lymphocytes (**A**), the percentage of CD4^+^ IFNγ^+^ T cells (**B**) and CD4^+^ T-bet^+^ T cells (**C**) within the peripheral blood T cells, and the percentage of CD4^+^ T-bet^+^ IFNγ^+^ T cells within the peripheral blood CD4^+^ T cells (**D**) in the indicated piglets. Data are presented as the mean ± SEM. *P < 0.05; **P < 0.01.

We assessed changes in the proportion of CD4^+^ T cells in peripheral blood by flow cytometry. An increase in the percentage of CD4^+^ T cells (Figure 5A; *P* = 0.001) was observed in group PDCoV compared with group CN. The percentage of CD4^+^ T-bet^+^ T cells (Figure 5C; *P* = 0.018) and CD4^+^ T-bet^+^ IFNγ^+^ T cells (Figure 5D; *P* = 0.030) were lower in group PDCoV than in group CN. No significant difference in the proportion of CD4^+^ IFNγ^+^ T cells was observed among three groups (Figure 5B).

### Administration of EP suppressed PDCoV-induced pro-inflammatory cytokines but promotes type I interferon

**Figure 6:**
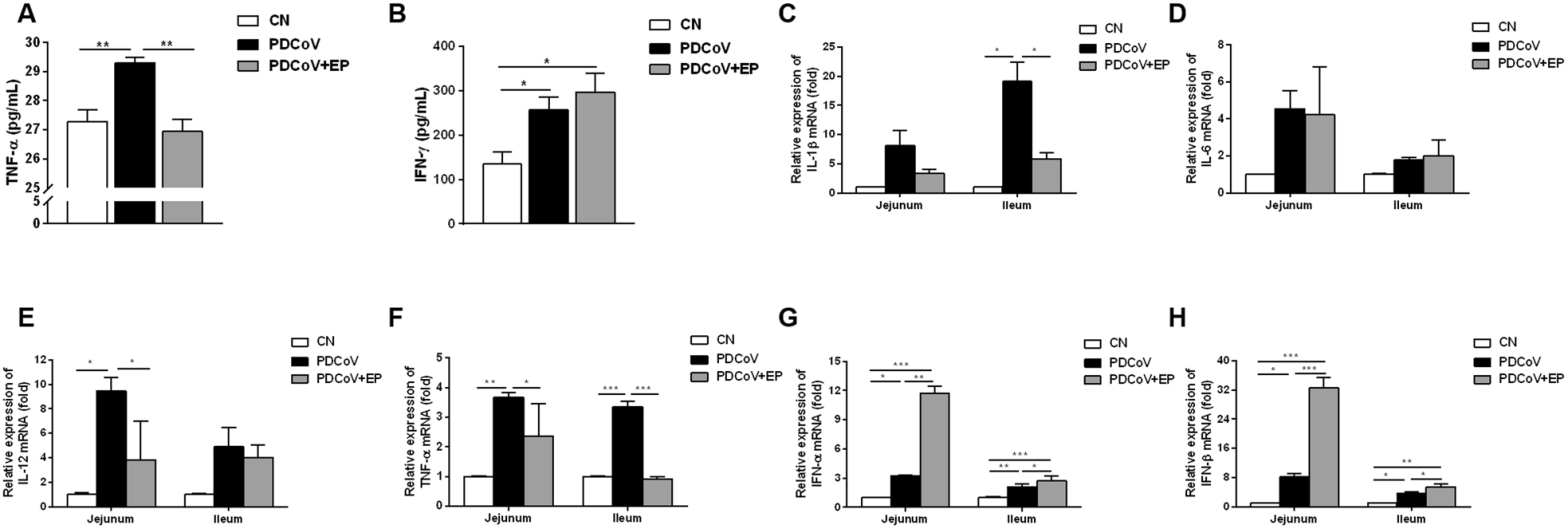
**Effect of EP on cytokines in piglets infected with PDCoV.** Peripheral blood samples of the indicated groups were collected at 5 dpi. The serum concentrations of TNF-α (**A**) and IFN-γ (**B**) were determined by ELISA. The relative expressions of IL-1β mRNA (C), IL-6 mRNA (D), IL-12 mRNA (**E**), TNF-α mRNA (**F**), IFN-α mRNA (**G**) and IFN-β mRNA (**H**) in jejunal and ileal tissues were assessed by RT-qPCR. Data are presented as the mean ± SEM. *P < 0.05; **P < 0.01; ***P < 0.001.

Elevation of serum TNF-α concentration was seen in group PDCoV (Figure 6A; *P* = 0.008) but not in group PDCoV + EP compared with group CN. PDCoV infection increased the amount of IFN-γ compared with group CN (Figure 6B; *P* = 0.041). No differences in the serum concentrations of IFN-γ were observed between groups PDCoV and PDCoV + EP. Increases in the mRNA expressions of ileal IL-1β (Figure 6C; *P* = 0.034), jejunal IL-12 (Figure 6E; *P* = 0.010), and ileal and jejunal TNF-α (Figure 6F; *P* = 0.003 and *P* < 0.001, respectively) were observed in group PDCoV but not in group PDCoV + EP, compared with group CN. INF-α (Figure 6G; *P* = 0.030 and *P* = 0.003, respectively) and IFN-β (Figure 6H; *P* = 0.019 and *P* = 0.017, respectively) mRNA expressions were upregulated in the jejunum and ileum of group PDCoV compared with group CN. Moreover, INF-α (Figure 6G; *P* = 0.001 and *P* = 0.012, respectively) and IFN-β (Figure 6H; *P* < 0.001 and *P* = 0.022, respectively) mRNA expressions were higher in the jejunum and ileum of group PDCoV + EP than that of group PDCoV. No differences in IL-6 mRNA expression were found in the small intestine among three groups (Figure 6D).

### Administration of EP suppresses PDCoV-induced activation of IκBα, NF-κB p65 and p38 in the small intestine

**Figure 7:**
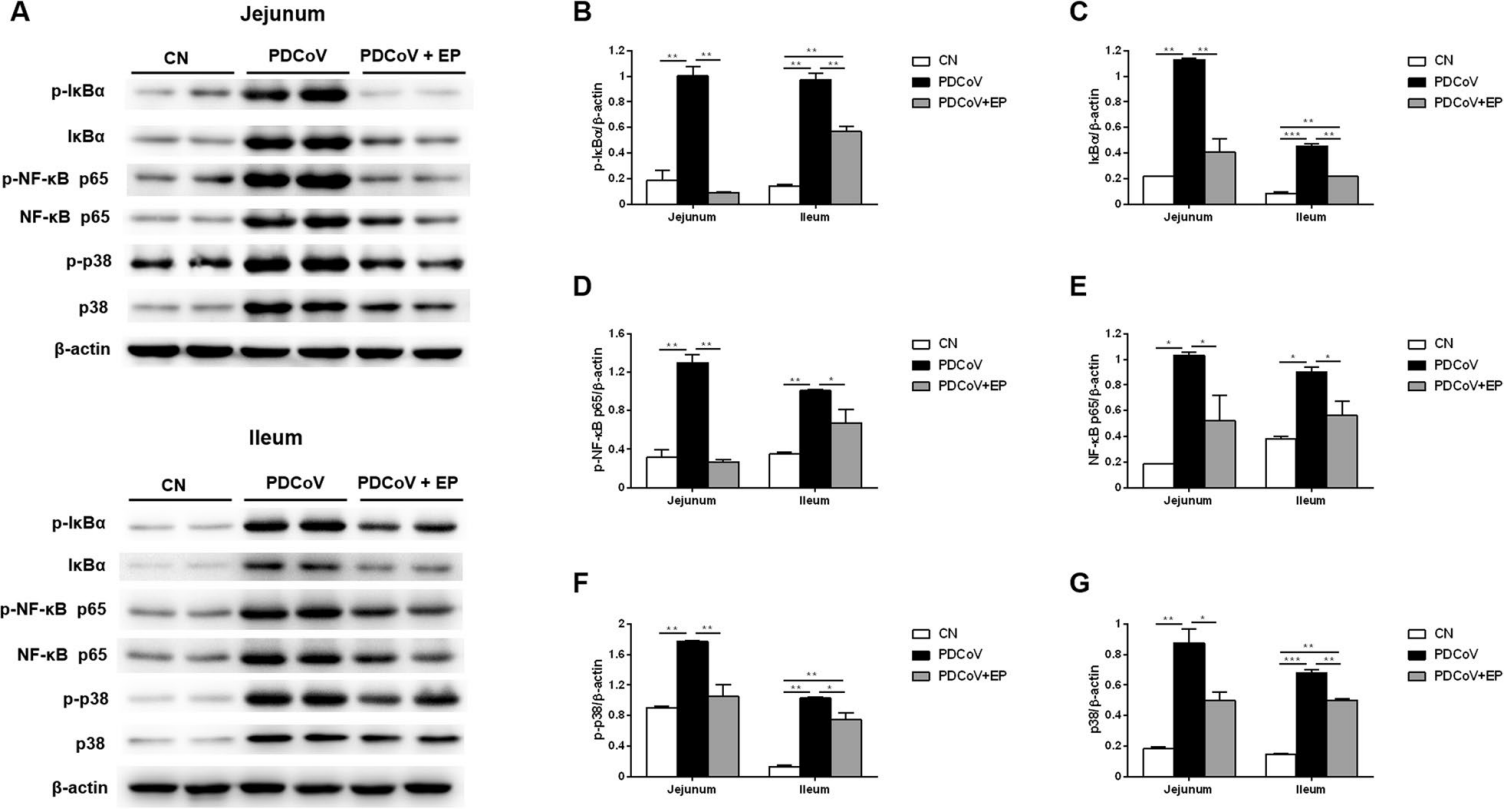
**Effect of EP on the NF-κB and p38/MAPK signaling pathways in the small intestine**. Intestinal tissue samples of the indicated groups were collected at 5 dpi. **A** Western blot analysis of proteins from jejunal and ileal tissues probed with the anti-p-IκBα, anti-IκBα, anti-p-NF-κB p65, anti-NF-κB p65, anti-p-p38 and anti-p38 antibody. **B**–**G**. Results are presented as the ratio of target protein band intensity to β-actin band intensity. Data are presented as the mean ± SEM. *P < 0.05; **P < 0.01; ***P < 0.001.

Western blot analysis demonstrated that PDCoV infection resulted in an increase in the expression of p-IκBα (Figures 7A, B; *P* = 0.006 and *P* = 0.001, respectively), p-NF-κB p65 (Figures 7A, D; *P* = 0.004 and *P* = 0.007, respectively) and p-p38 (Figures 7A, F; *P* = 0.005 and *P* = 0.001, respectively) in the jejunum and ileum compared with group CN. However, the phosphorylation levels of IκBα (Figures 7A, B; *P* = 0.004 and *P* = 0.007, respectively), NF-κB p65 (Figures 7A, D; *P* = 0.003 and *P* = 0.049, respectively) and p38 (Figures 7A, F; *P* = 0.009 and *P* = 0.033, respectively) in the jejunum and ileum of group PDCoV + EP were obviously lower than those of group PDCoV, indicating that EP can alleviate the activation of the NF-κB and p38/MAPK signaling pathways caused by PDCoV.

### EP inhibits PDCoV replication and alleviates PDCoV-induced apoptosis via p38/MAPK signaling pathway

**Figure 8:**
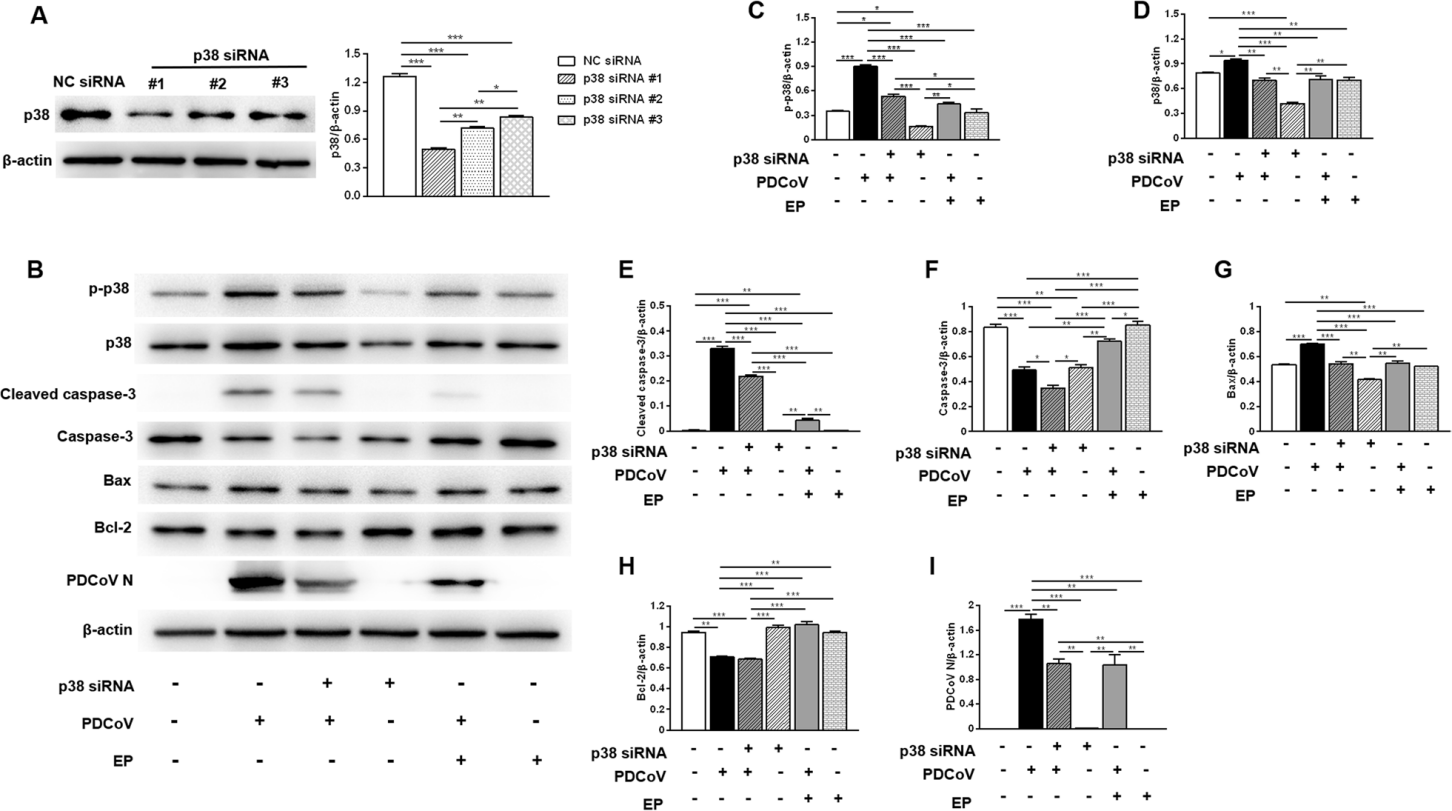
**Role of p38 in inhibiting PDCoV replication and alleviating PDCoV-induced apoptosis by EP in LLC-PK1 cells.** **A** The efficiency of p38 siRNA was evaluated by Western blot. LLC-PK1 cells were transfected with the indicated siRNA. At 24 h post-transfection, the expression of p38 was analyzed by Western blot. Results were presented as the ratio of p38 band intensity to β-actin band intensity. **B** LLC-PK1 cells grown in 6-well plates were transfected with p38 siRNA #1 or NC siRNA for 24 h, and then infected with 2 MOI PDCoV or mock-infected in the absence or presence EP (150 μM). After PDCoV adsorption for 1 h, the cells were further cultured in fresh medium in the absence or presence EP (150 μM). Western blot analysis of proteins from indicated LLC-PK1 cells probed with the anti-p-p38, anti-p38, anti-cleaved caspase-3, anti-caspase-3, anti-Bax, anti-Bcl-2 and anti-PDCoV N antibody. **C**–**I** Results are presented as the ratio of target protein band intensity to β-actin band intensity. Data are presented as the mean ± SEM. *P < 0.05; **P < 0.01; ***P < 0.001.

We used siRNA-mediated knockdown to further address the role of p38 in inhibiting PDCoV replication and alleviating PDCoV-induced apoptosis by EP in LLC-PK1 cells. The siRNA targeting p38 apparently reduced the expression of p38 (Figure 8A), and p38 siRNA #1 was used for subsequent experiments. Compared with the uninfected cells, cells infected with PDCoV only had higher expression levels of p-p38 (Figures 8B, C), cleaved-caspase-3 (Figures 8B, E) and bax (Figures 8B, G). However, the expression levels of cleaved-caspase-3 (Figures 8B, E), bax (Figures 8B, G) and PDCoV N (Figures 8B, I) in cells transfected with p38 siRNA before PDCoV infection were lower than those of cells infected with PDCoV only. These results suggest that PDCoV may activate p38 to facilitate its replication and induce apoptosis. Compared with cells infected with PDCoV only, cells infected with PDCoV in the presence of EP showed significantly lower expression of PDCoV N (Figures 8B, I), p-p38 (Figures 8B, C), cleaved-caspase-3 (Figures 8B, E) and bax (Figures 8B, G), implying that EP may inhibit PDCoV replication and alleviate PDCoV-induced apoptosis by suppressing p38 activation caused by PDCoV.

## Discussion

As PDCoV spreads around the world, placing strain on the economy and public health, the prevention and treatment of PDCoV is clearly important. Until now, there has been no report about PDCoV vaccines, and only few reports about anti-PDCoV drugs. Lithium chloride and diammonium glycyrrhizinate (DG) were reported to inhibit PDCoV replication at the early stage in LLC-PK1 cells, and DG also inhibited virus attachment to the cells [23]. Rhodanine derivative LJ001 showed antiviral activity against PDCoV infection in ST cells [24]. Besides, PDCoV was shown to be susceptible to remdesivir treatment in Huh7 cells [25]. However, there has been no record of effective drugs for the treatment of PDCoV infection in vivo. Our previous study revealed that EP from the mushroom *C. volvatus* has efficient anti-PDCoV properties in LLC-PK1 and ST cells, targeting multiple stages of the PDCoV life cycle, including attachment, entry, and the early and middle stages of the post-entry stage, and even has a virucidal effect against PDCoV [9]. The present results demonstrated that oral administration of EP diminished the pathological manifestation caused by PDCoV infection and reduced viral load in piglets, showing the promise of EP in the treatment of PDCoV, and making up the vacancy in clinical research on anti-PDCOV drugs.

The intestinal barrier acts as a semi-permeable barrier allowing the absorption of different molecules, such as water or nutrients [26], and plays a leading role in regulating the immune system through recognizing microorganisms [27]. It is maintained by two key mechanisms: epithelial cell homeostasis to regulate cell numbers forming the barrier and homotypic junctional complexes to regulate paracellular permeability across the barrier [28]. Intestinal epithelial cell homeostasis is established by equilibrium between cell proliferation and cell death. Dysregulated or excessive epithelial cell death is associated with the destruction of barrier integrity and leakage across the paracellular space. Histopathologic analysis revealed that the jejunal and ileal tissues infected with PDCoV exhibited enterocyte attenuation. The massive loss of enterocytes destroyed the intestinal barrier, hampered the absorption and digestion of nutrients and electrolytes in the small intestines, causing malabsorptive and dyspepsia diarrhea that consequently led to fatal dehydration in piglets. Next, we found that PDCoV infection resulted in severe apoptosis in the jejunum and ileum. This is consistent with one previous study on another CoV, PEDV, causing intestinal apoptosis [29], but contrary to another study which showed that PDCoV did not induce apoptosis in the infected intestinal enterocytes in vivo [30]. The difference in results may be due to the age of piglets and the time of necropsy. Administration of EP reduced PDCoV-induced apoptosis and was beneficial to maintain the integrity intestinal barrier.

TJ proteins connect the intestinal epithelial cells and regulate the paracellular permeability [28]. The destruction of these proteins may cause the damage of intestinal barrier. One previous study showed that impaired TJs in intestinal epithelium of patients with human immunodeficiency virus contributed to intestinal barrier dysfunction, resulting in severe, chronic diarrhea [31]. Administration of EP recovered PDCoV-decreased jejunal and ileal ZO-1 expression and improved jejunal claudin-1 expression. Taken together, EP’s protection of intestine from PDCoV infection may be attributed to its ability to inhibit apoptosis and avoid TJ protein loss.

During viral infection, CD4^+^ T cells can provide effective immunity protection through direct effector function and by helping other leukocytes to maximize the protective activities [12]. Our present results demonstrated that the proportion of CD4^+^ T cells in peripheral blood increased after PDCoV infection, which is similar to PEDV infection [32]. Besides, PDCoV infection led to a reduction in the percentage of CD4^+^ T-bet^+^ IFNγ^+^ T cells in peripheral blood. To our knowledge, this is the first report to prove the impact of PDCoV infection on CD4^+^ T cells, which provides further insights for understanding the immune response to PDCoV.

Cytokines are crucial in immune defense and pathogenesis during viral infections. Administration of EP promoted type I interferon expression, promoting its antiviral activity against PDCoV. In addition, PDCoV infection increased TNF-α concentration in the serum and the mRNA levels of IL-1β, IL-12 and TNF-α in the small intestine. These increased pro-inflammatory cytokines may coordinate and activate adaptive immune responses. However, their excessive release may also mediate immunopathology. For example, acute lung injury caused by severe acute respiratory syndrome coronavirus (SARS-CoV) or Middle Eastern respiratory syndrome coronavirus (MERS-CoV) infection is closely related to the elevated pro-inflammatory cytokine responses, such as IL-6, IL-12 and TNF-α [33]. Intensive immunosuppression therapy with high dose glucocorticoids, followed by an IL-6 receptor antagonist reduced the mortality as a result of severe acute respiratory syndrome coronavirus 2 (SARS-CoV-2)-associated cytokine storm syndrome [34]. Here, administration of EP reduced PDCoV-induced pro-inflammatory cytokines to similar levels as group CN, implying that EP has an immunomodulatory effect during PDCoV infection. NF-κB is one of the key transcription factors that regulate the production of pro-inflammatory cytokines. Administration of EP suppressed PDCoV-induced activation of NF-kB signaling pathway, which in turn reduced the amount of pro-inflammatory cytokines.

Virus relies on the host cell machinery for effective replication. MAPK signaling pathways are reported to be manipulated by CoVs to facilitate their replication. For example, inhibition of p38 or JNK1/2 resulted in a significant reduction in PEDV RNA synthesis, protein expression and progeny release [35]. In addition, the p38/MAPK signaling pathway can convert extracellular stimuli into a wide range of cellular responses, including apoptosis [19]. Here, we found that knockdown of p38 decreased PDCoV replication and alleviated PDCoV-induced apoptosis, implying that PDCoV might activate the p38/MAPK signaling pathway to facilitate its replication and induce apoptosis. EP decreased PDCoV-induced activation of p38, which may contribute to its inhibition on PDCoV replication and alleviation of apoptosis induced by PDCoV.

In conclusion, our study in a piglet model indicates that oral administration of EP confers a degree of protection against PDCoV infection, providing potential insights for the clinical prevention and treatment of PDCoV.

## Abbreviations

PDCoV: Porcine deltacoronavirus
CoV: Coronavirus
EP: Ergosterol peroxide
TJ: Tight junction
NF-κB: Nuclear factor-κB
IκB: Inhibitor of NF-κB
IL: Interleukin
TNF-α: Tumor necrosis factor-α
IFN: Interferon
TCID_50_: 50% Tissue culture infection dose
MAPK: Mitogen-activated protein kinase
JNK: Janus kinase
PEDV: Porcine epidemic diarrhea virus
RT-qPCR: Quantitative reverse transcription PCR
ELISA: Enzyme-linked immunosorbent assay
TUNEL: Terminal deoxynucleotidyl transferase-mediated dUTP nick end labelling
